# Successful treatment of telomeropathy‐related interstitial lung disease with immunosuppression and danazol

**DOI:** 10.1002/rcr2.607

**Published:** 2020-06-25

**Authors:** Daniel C. Chambers, Viviana P. Lutzky, Simon H. Apte, David Godbolt, John Feenstra, John Mackintosh

**Affiliations:** ^1^ Qld Lung Transplant Service The Prince Charles Hospital Brisbane QLD Australia; ^2^ School of Clinical Medicine The University of Queensland Brisbane QLD Australia; ^3^ Department of Pathology The Prince Charles Hospital Brisbane QLD Australia

**Keywords:** Danazol, interstitial lung disease, non‐specific interstitial pneumonia, telomere

## Abstract

We report the case of a 42‐year‐old female with a history of finger clubbing which improved during pregnancy, a history of unexplained hepatosplenomegaly, and subsequent non‐specific interstitial pneumonia with respiratory failure. Given a personal and family history of early greying of the hair, the peripheral blood monocyte telomere length was measured and was confirmed to be <1st centile, explaining the multiorgan presentation. She was treated with prednisolone, mycophenolate mofetil, and the synthetic androgen danazol with a dramatic improvement in respiratory failure and lung function. After 18 months of danazol treatment, the peripheral blood monocyte telomere length had returned to the normal range.

## Graphical abstract

Genomic contributors to the pathogenesis of interstitial lung disease are increasingly recognized. This case report describes dramatic clinical response to a combination of immunosuppression and danazol in a patient with non‐specific interstitial pneumonia associated with very short telomeres.

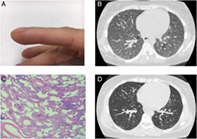

## Introduction

In a substantial minority of patients, interstitial lung disease (ILD) is related to genetic mutations, with the most common mutations found in telomere maintenance genes. Although initially recognized in patients with idiopathic pulmonary fibrosis, mutations in telomere maintenance genes and telomere shortening are found in all ILDs [1]. Telomeres are long lengths of DNA which prevent chromosomal shortening during cell division. Chromosome protection is lost when telomere length is reduced leading to cellular senescence. Telomere shortening is a normal ageing process that is regulated by telomerase, an enzyme which acts to preserve telomere length. Mutations in telomere maintenance genes (including TERT, TERC, and others) cause premature telomere attrition. Although it is now recognized that telomere shortening is fundamental to ILD pathogenesis in some patients, the cellular events that lead to inflammation and fibrosis are poorly understood. Patients with a telomeropathy underlying their ILD often present with extrapulmonary organ dysfunction, including bone marrow failure.

The TERT promoter is sex steroid responsive and the synthetic androgen danazol has been shown to lengthen telomeres [2]. There are pilot data suggesting that danazol may be associated with stabilization of lung function for up to two years in patients with haematological disease related to telomere shortening [3].

## Case Report

At the age of 34 years, a medical friend noticed finger clubbing and recommended clinical review. At the time, the patient had been well with no respiratory symptoms. The past medical history included obesity and endometriosis which was untreated. After an extensive evaluation, the only abnormality identified was hepatosplenomegaly, with a liver span of 18 cm and spleen of 14 cm. No cause was able to be identified; however, after significant weight loss, the hepatosplenomegaly improved. The finger clubbing persisted, but then resolved completely during a pregnancy at the age of 37 years.

**Figure 1:**
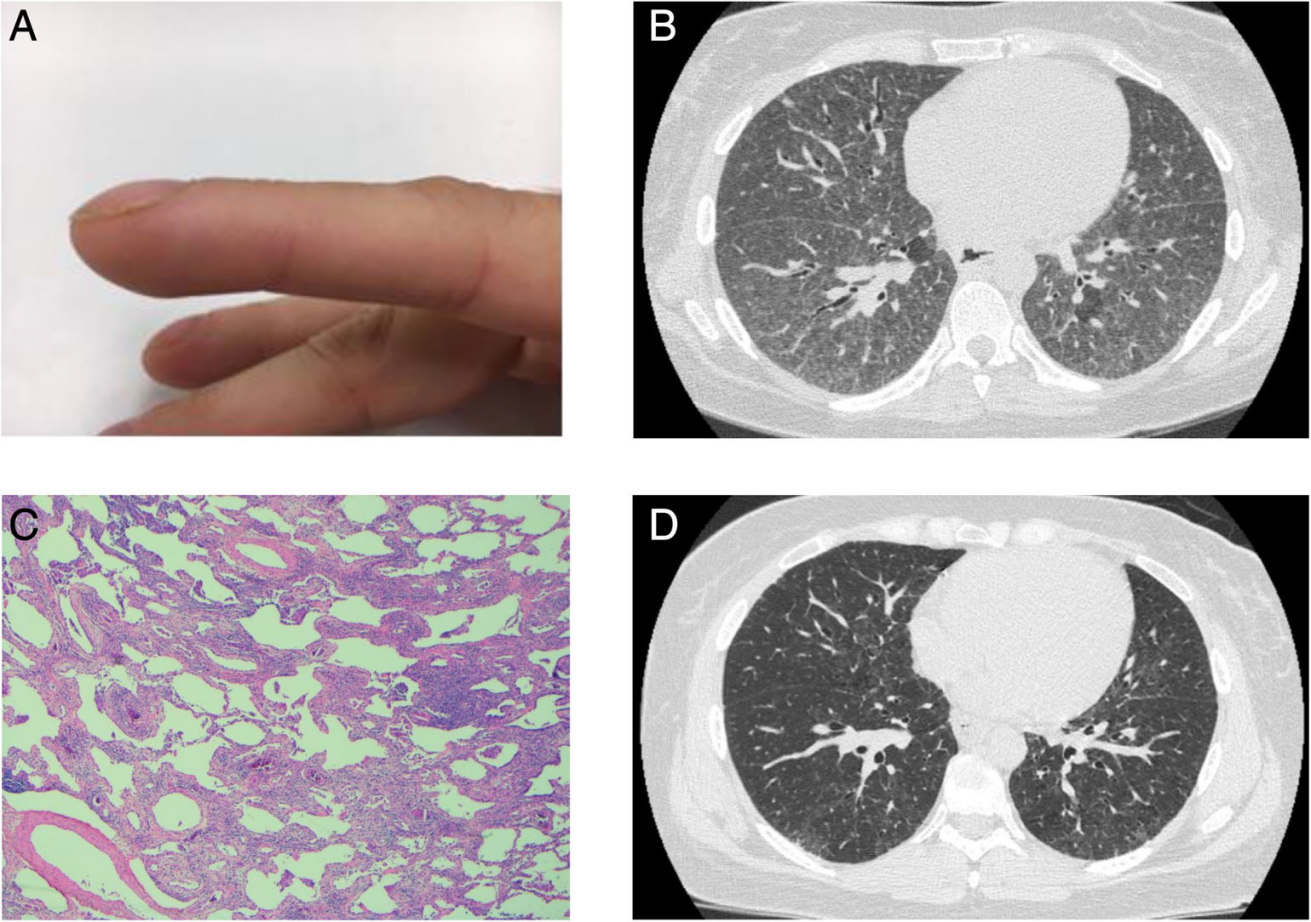
(A) The patient developed finger clubbing which resolved during pregnancy. (B) A high‐resolution computed tomography (HRCT) of the chest reveals extensive, bilateral reticular nodular opacification, and associated wide‐spread ground glass with no significant zonal predominance. There are small islands of relative lucency consistent with mosaicism. (C) An open lung biopsy demonstrates a mild to moderate diffuse interstitial lymphocyte infiltrate with interstitial fibrosis devoid of spatial and temporal heterogeneity or architectural remodelling (haematoxylin and eosin (H&E): 40×), consistent with non‐specific interstitial pneumonia. (D) A follow‐up HRCT obtained two years later, after treatment with immunosuppression and danazol, demonstrates significant improvement.

At the age of 39 years, breathlessness and a dry cough developed, and the finger clubbing returned. Over the subsequent year, respiratory failure and marked hypoxaemia developed. The chest examination revealed widespread fine crepitations. Lung function testing confirmed severe lung disease with a forced vital capacity of 2.05 L (54% of predicted) and a diffusing capacity for carbon monoxide of 5.01 mL/min/mmHg (21% of predicted). Chest imaging revealed an unclassifiable ILD with widespread ground‐glass opacification, reticulation, and focal mosaicism (Fig. 1). There was no history of exposure to antigens which may trigger hypersensitivity pneumonitis, no clinical evidence of a connective tissue disorder, and anti‐nuclear antibody and extractable nuclear antigen testing was negative, as was a myositis antibody panel and creatine kinase. The full blood count was normal, with normal platelets and mean corpuscular volume. An open lung biopsy (Fig. 1) confirmed non‐specific interstitial pneumonia.

Given the history of unexplained extrapulmonary organ dysfunction, rapidly progressing ILD and, on further questioning, a personal (age 21 years) and family history of premature greying of the hair, a telomeropathy was suspected. Peripheral blood monocyte telomere length was measured using flow cytometry‐fluorescence in situ hybridization (flow‐FISH) and was found to be <1st centile for age.

**Figure 2:**
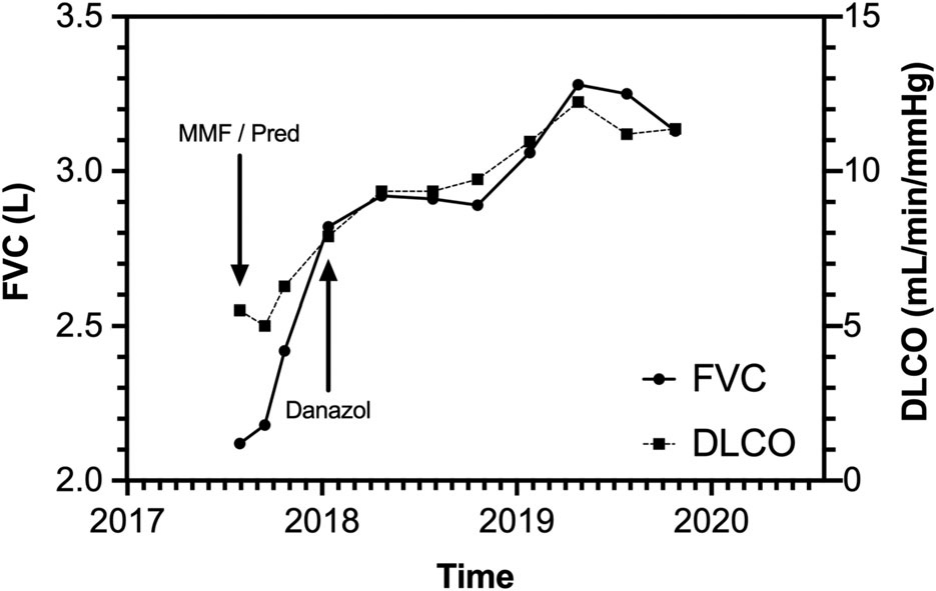
Lung function trends over 2.5 years. The forced vital capacity (FVC, L) and diffusing capacity (DLCO, mL/min/mmHg) are shown, as are the timepoints at which immunosuppression and danazol were commenced.

Immunosuppression with prednisolone and mycophenolate mofetil was commenced. After four months of treatment, there had been a modest improvement in respiratory symptoms and lung function tests (forced vital capacity increased to 2.82 L and the diffusing capacity increased to 7.89 mL/min/mmHg). A transplant assessment was arranged on the basis of persistent severe lung disease and the patient's young age. Given the reports of telomere lengthening with danazol and the history of improvement in clubbing with pregnancy suggesting that the underlying pathology may be responsive to sex steroid, the possibility of trialling danazol in order to avoid transplantation was discussed. After the exclusion of pregnancy, danazol was commenced at 400 mg twice daily. Danazol was well tolerated with no side effects and subsequently there was a further significant improvement in functional status so that the patient became asymptomatic, the radiological appearance improved (Fig. 1), lung function tests returned to near normal (Fig. 2), and immunosuppression could be weaned. After 18 months of treatment with danazol, the peripheral blood monocyte telomere length had returned to the normal range (>10th centile).

## Discussion

The genetic basis of not only idiopathic pulmonary fibrosis, but also many ILDs has recently been recognized, with a substantial minority of patients having mutations in telomere maintenance genes or short telomeres without an identifiable mutation [1]. This patient group may have a history of premature greying of the hair, thrombocytopaenia, macrocytosis, and other haematological abnormalities [4], and experiences worse survival, with the genetics providing more accurate information regarding prognosis than multidisciplinary phenotyping [5]. With the advent of antifibrotic treatments for idiopathic pulmonary fibrosis and recognition of their efficacy in a wide range of ILDs, attention is now turning to more fundamental causes of ILD and fibrosis with the aim of personalizing care. Already, it is apparent that understanding the genetic causes of ILD in any particular individual can impact positively on treatment choice. Precision medicine is hence the next frontier for ILD management.

Here, we describe a dramatic clinical, radiological, physiological, and genetic response to a combination of immunosuppression and synthetic androgen treatment in a patient with severe telomere‐related progressive ILD. Although clinical trials will be needed to determine if androgen therapy is effective for telomere‐related ILDs, the case highlights the importance of understanding the fundamental causes of disease in order to make appropriate management decisions and speaks to the need for the development of well‐designed pharmacological agents aimed at correcting disease‐causing genetic defects. This approach has already paid huge dividends for patients with cystic fibrosis. It is hoped that in coming years, similar improvements will be seen for patients with ILDs.

### Disclosure Statement

Appropriate written informed consent was obtained for publication of this case report and accompanying images.

## References

[1] Snetselaar R , van Moorsel CHM , Kazemier KM , et al. 2015 Telomere length in interstitial lung diseases. Chest 148:1011–1018. 25973743 10.1378/chest.14-3078

[2] Calado RT , Yewdell WT , Wilkerson KL , et al. 2009 Sex hormones, acting on the TERT gene, increase telomerase activity in human primary hematopoietic cells. Blood 114:2236–2243. 19561322 10.1182/blood-2008-09-178871 PMC2745844

[3] Townsley DM , Dumitriu B , Liu D , et al. 2016 Danazol treatment for telomere diseases. N. Engl. J. Med. 374:1922–1931. 27192671 10.1056/NEJMoa1515319 PMC4968696

[4] Chambers DC , Clarke BE , McGaughran J , et al. 2012 Lung fibrosis, premature graying, and macrocytosis. Am. J. Respir. Crit. Care Med. 186:e8–e9. 22942352 10.1164/rccm.201112-2175IM PMC3443804

[5] Newton CA , Oldham JM , Ley B , et al. 2019 Telomere length and genetic variant associations with interstitial lung disease progression and survival. Eur. Respir. J. 53:1801641. 30635297 10.1183/13993003.01641-2018 PMC6612265

